# Mitochondrial respiratory chain is involved in insulin-stimulated hydrogen peroxide production and plays an integral role in insulin receptor autophosphorylation in neurons

**DOI:** 10.1186/1471-2202-8-84

**Published:** 2007-10-08

**Authors:** Tatiana P Storozhevykh, Yana E Senilova, Nadezhda A Persiyantseva, Vsevolod G Pinelis, Igor A Pomytkin

**Affiliations:** 1 Scientific Centre for Children's Health, RAMS, Lomonosovsky prospect 2/62, 119991, Moscow, Russia; 2 Biosignal Ltd., M. Gruzinskaya 29-153, 123557, Moscow, Russia

## Abstract

**Background:**

Accumulated evidence suggests that hydrogen peroxide (H_2_O_2_) generated in cells during insulin stimulation plays an integral role in insulin receptor signal transduction. The role of insulin-induced H_2_O_2 _in neuronal insulin receptor activation and the origin of insulin-induced H_2_O_2 _in neurons remain unclear. The aim of the present study is to test the following hypotheses (1) whether insulin-induced H_2_O_2 _is required for insulin receptor autophosphorylation in neurons, and (2) whether mitochondrial respiratory chain is involved in insulin-stimulated H_2_O_2 _production, thus playing an integral role in insulin receptor autophosphorylation in neurons.

**Results:**

Insulin stimulation elicited rapid insulin receptor autophosphorylation accompanied by an increase in H_2_O_2 _release from cultured cerebellar granule neurons (CGN). N-acetylcysteine (NAC), a H_2_O_2 _scavenger, inhibited both insulin-stimulated H_2_O_2 _release and insulin-stimulated autophosphorylation of insulin receptor. Inhibitors of respiratory chain-mediated H_2_O_2 _production, malonate and carbonyl cyanide-4-(trifluoromethoxy)-phenylhydrazone (FCCP), inhibited both insulin-stimulated H_2_O_2 _release from neurons and insulin-stimulated autophosphorylation of insulin receptor. Dicholine salt of succinic acid, a respiratory substrate, significantly enhanced the effect of suboptimal insulin concentration on the insulin receptor autophosphorylation in CGN.

**Conclusion:**

Results of the present study suggest that insulin-induced H_2_O_2 _is required for the enhancement of insulin receptor autophosphorylation in neurons. The mitochondrial respiratory chain is involved in insulin-stimulated H_2_O_2 _production, thus playing an integral role in the insulin receptor autophosphorylation in neurons.

## Background

Accumulated evidence suggests that hydrogen peroxide (H_2_O_2_) generated in cells during insulin stimulation plays an integral role in insulin receptor signal transduction [1-4]. Specific molecular targets of H_2_O_2 _identified to date include the insulin receptor kinase [5-7], protein tyrosine phosphatases (PTP) [8-11], and the lipid phosphatase PTEN [12], whose activity is modified via oxidative reactions with H_2_O_2_. Two distinct insulin-sensitive cellular H_2_O_2 _sources have been identified. A membrane-bound NADPH-oxidase is involved in insulin-induced H_2_O_2 _production in adipocytes [13-17] and vascular smooth muscle cells [18,19]. The mitochondrial respiratory chain is implicated in insulin-induced H_2_O_2 _generation in liver and heart [20,21]. There are experimental data that insulin-induced reactive oxygen species (ROS) and H_2_O_2 _play a role in the activation of insulin signaling in neuroblastomas [12,22]. However, the role of insulin-induced H_2_O_2 _in neuronal insulin receptor activation and the origin of insulin-induced H_2_O_2 _in neurons remain unclear.

The aim of the present study is to test the following hypotheses (1) whether insulin-induced H_2_O_2 _is required for insulin receptor autophosphorylation in neurons, and (2) whether mitochondrial respiratory chain is involved in insulin-stimulated H_2_O_2 _production, thus playing an integral role in insulin receptor autophosphorylation in neurons.

## Results

### Insulin-induced H_2_O_2 _is required for the enhancement of the insulin receptor autophosphorylation in neurons

**Figure 1:**
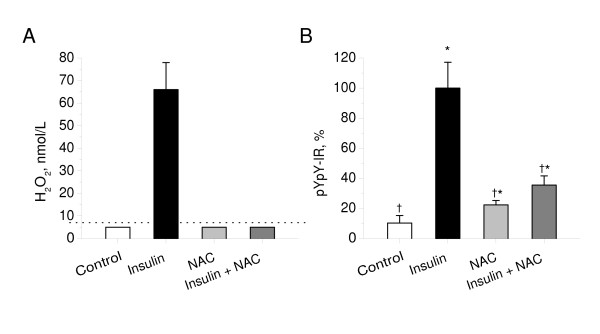
**Effect of N-acetylcysteine on Insulin-stimulated H_2_O_2 _production and the insulin receptor autophosphorylation in cerebellar granule neurons**. A: CGN cultures were pre-incubated for 30 min in the absence or presence of N-acetylcysteine (5 mmol/l) in Hepes-buffered salt solution and then exposed to insulin (100 nmol/L). H_2_O_2 _release from cultures for 1 min was measured as described in Materials and Methods. Results were normalized by cell density. Columns represent the means ± SD of H_2_O_2 _values obtained from five to nine cultures. Dotted line represents a detection limit of the assay (7 nmol/L). B: CGN cultures were pre-incubated for 30 min in the absence or presence of N-acetylcysteine (5 mmol/l) in Hepes-buffered salt solution and then exposed to insulin (100 nmol/L) for 20 min. Autophosphorylation of insulin receptor was measured as described in Materials and Methods. In each experiment, amount of phosphorylated insulin receptor β-subunit (pYpY-IR) was normalized to total amount of insulin receptor β-subunit and expressed as a percentage of the response produced to 100 nmol/L insulin. Columns represent the means ± SD of pYpY-IR values obtained from four to nine culture dishes. *P < 0.05 vs. control.^†^P < 0.05 vs. insulin.

To examine whether insulin stimulates H_2_O_2 _production in cultured cerebellar granule neurons (CGN), we measured H_2_O_2 _accumulation for 1 min in the incubation medium of CGN cultures, in the absence or presence of insulin. For H_2_O_2 _detection, an extremely sensitive assay based on fluorescence of resorufin, a product of a 1:1 stoichiometric reaction of Amplex red dye with H_2_O_2_, was used. As shown in Figure 1A, insulin stimulation elicited a marked increase in H_2_O_2 _release from CGN to a level of 66 ± 12 nmol/L, although the basal H_2_O_2 _release from CGN cultures was below the assay detection limit (< 7 nmol/L).

To determine whether insulin-induced H_2_O_2 _is involved in the enhancement of insulin receptor autophosphorylation, we next studied the effects of N-acetylcysteine (NAC), a H_2_O_2 _scavenger, on the insulin-stimulated autophosphorylation of the insulin receptor in CGN. As shown in Figure 1A, the pre-incubation of CGN with NAC abolished the insulin-stimulated H_2_O_2 _release from cells to undetectable levels (<7 nmol/L), indicating that NAC is a potent scavenger of insulin-induced H_2_O_2 _under these experimental conditions. Figure 1B shows that pre-incubation of CGN with NAC resulted in the significant inhibition of insulin-stimulated insulin receptor autophosphorylation (P < 1e-6 vs. insulin). These results suggest that insulin-induced H_2_O_2 _is required for the enhancement of insulin receptor autophosphorylation in neurons.

### The mitochondrial respiratory chain is involved in insulin-stimulated H_2_O_2 _production, thus playing an integral role in the insulin receptor autophosphorylation in neurons

**Figure 2:**
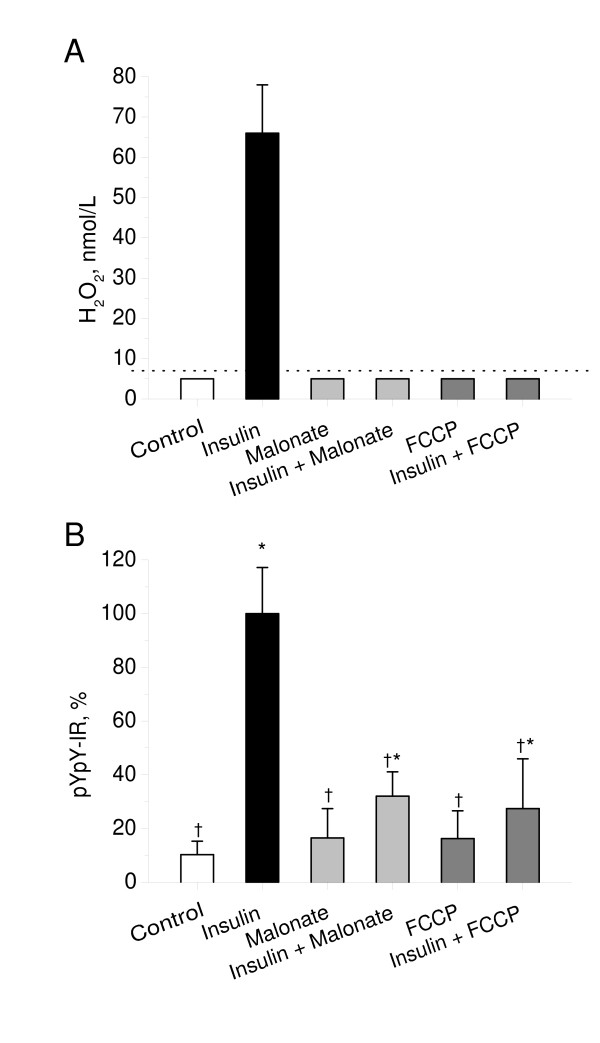
**Effects of malonate and FCCP on insulin-stimulated H_2_O_2 _production and insulin receptor autophosphorylation in cerebellar granule neurons**. A: CGN cultures were pre-incubated for 30 min in Hepes-buffered salt solution and then exposed to insulin (100 nmol/L) in the absence or presence of malonate (2 mmol/L) or FCCP (0.5 μmol/L). H_2_O_2 _release from cultures for 1 min was measured as described in Materials and Methods. Results were normalized by cell density. Columns represent the means ± SD of H_2_O_2 _values obtained from five to nine cultures. Dotted line represents a detection limit of the assay (7 nmol/L). B: CGN cultures were pre-incubated for 30 min in Hepes-buffered salt solution and then exposed to insulin (100 nmol/L) for 20 min. Malonate (2 mmol/l) or FCCP (0.5 μmol/L) were added to cultures 5 min before the insulin exposure. Autophosphorylation of insulin receptor was measured as described in Materials and Methods. In each experiment, amount of phosphorylated insulin receptor β-subunit (pYpY-IR) was normalized to total amount of insulin receptor β-subunit and expressed as a percentage of the response produced to 100 nmol/L insulin. Columns represent the means ± SD of pYpY-IR values obtained from four to nine culture dishes. *P < 0.05 vs. control.^†^P < 0.05 vs. insulin.

To reveal whether mitochondrial respiratory chain is involved in the insulin-stimulated H_2_O_2 _production in neurons, we measured H_2_O_2 _release from CGN cultures in the absence or presence of insulin and two inhibitors of respiratory chain-mediated H_2_O_2 _production, malonate and carbonyl cyanide-4-(trifluoromethoxy)-phenylhydrazone (FCCP). As shown in Figure 2A, both malonate and FCCP completely abolished insulin-stimulated H_2_O_2 _release from CGN. Both malonate and FCCP had no effect on basal H_2_O_2 _production in CGN. These data indicate that mitochondrial respiratory chain is involved in insulin-stimulated H_2_O_2 _generation in neurons.

**Figure 3:**
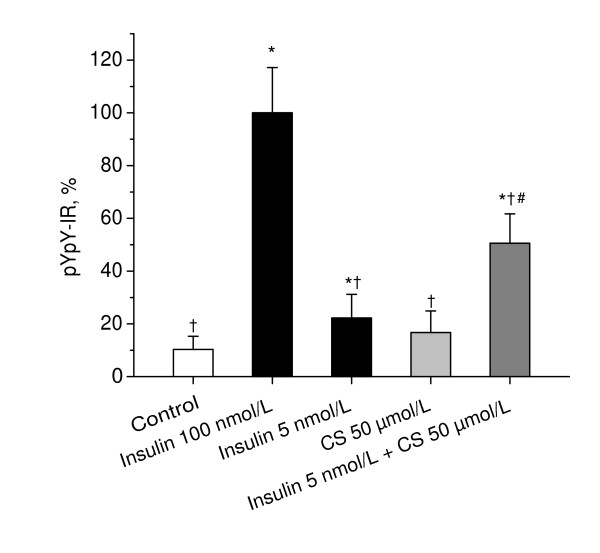
**Effects of dicholine salt of succinic acid on insulin-stimulated insulin receptor autophosphorylation in cerebellar granule neurons**. CGN cultures were pre-incubated for 30 min in Hepes-buffered salt solution and then exposed to insulin (100 nmol/L), insulin (5 nmol/L), CS (50 μmol/L), or a combination of insulin (5 nmol/L) and CS (50 μmol/L) for 20 min. Autophosphorylation of insulin receptor was measured as described in Materials and Methods. In each experiment, amount of phosphorylated insulin receptor β-subunit (pYpY-IR) was normalized to total amount of insulin receptor β-subunit and expressed as a percentage of the response produced to 100 nmol/L insulin. Columns represent the means ± SD of pYpY-IR values obtained from four to nine culture dishes. *P < 0.05 vs. control.^†^P < 0.05 vs. insulin (100 nmol/L). ^#^P < 0.05 vs. insulin (5 nmol/L).

To examine whether mitochondrial respiratory chain is involved in insulin receptor autophosphorylation in neurons, we assessed the effects of inhibitors of respiratory chain-mediated H_2_O_2 _production, malonate and FCCP, and a respiratory substrate, dicholine salt of succinic acid (CS), on the insulin-stimulated autophosphorylation of the insulin receptor in primary CGN cultures. Whereas by itself, malonate, FCCP, and CS had no effect on the basal autophosphorylation of the insulin receptor, they significantly influenced the insulin-stimulated autophosphorylation of insulin receptor. As shown in Figure 2B, both malonate and FCCP significantly inhibited the insulin-stimulated autophosphorylation of the insulin receptor (P < 1e-5 vs. insulin). Figure 3 shows that CS significantly enhanced the effect of suboptimal concentration of 5 nmol/L insulin on insulin receptor autophosphorylation (P < 0.001 vs. insulin). These data suggest that mitochondrial respiratory chain plays an integral role in insulin receptor autophosphorylation in neurons.

## Discussion

Insulin signaling requires the autophosphorylation of the insulin receptor kinase at tyrosine residues in the activation loop of the kinase domain [23-28]. Upon autophosphorylation, the receptor undergoes a major conformational change resulting in unrestricted access of protein substrates and ATP to the kinase active site and an approximate two-order increase in kinase activity [29-31]. In the present study, we demonstrate that N-acetylcysteine, the H_2_O_2 _scavenger, inhibits both insulin-stimulated H_2_O_2 _generation and insulin-stimulated insulin receptor autophosphorylation in CGN. These results suggest that insulin-induced H_2_O_2 _is required for the enhancement of insulin receptor autophosphorylation in neurons.

We found that mitochondrial respiratory chain is involved in insulin-stimulated H_2_O_2 _production, thus playing an integral role in insulin receptor autophosphorylation in neurons. Mitochondrial respiration is a major cellular source of H_2_O_2 _that may convert up to 2% of total oxygen consumption into H_2_O_2 _in state 4 respiration (oxygen consumption in the absence of ADP) with succinate as a respiratory substrate [32]. Among other respiratory substrates, the complex II substrate succinate provides the highest rates of mitochondrial H_2_O_2 _generation in brain mitochondria [33]. Molecular mechanisms of H_2_O_2 _production in mitochondria are the subject of intense ongoing research. The respiratory chain reduces oxygen to superoxide anion, which dismutates to H_2_O_2 _spontaneously or by the action of superoxide dismutase [34]. Although mitochondria produce H_2_O_2 _in all metabolic states, high mitochondrial membrane potential (ΔΨ) characteristic to the resting (State 4) respiration significantly promotes H_2_O_2 _generation [33,35]. Substances that decrease (ΔΨ), e.g. malonate and FCCP, inhibit H_2_O_2 _generation [33,35-37]. In the present study, we demonstrate that inhibitors of respiratory chain-mediated H_2_O_2 _production, malonate and the protonophore FCCP, inhibit both insulin-induced H_2_O_2 _generation and insulin-stimulated receptor autophosphorylation in neurons. The respiratory substrate succinate, taken in form of dicholine salt of succinic acid, significantly enhances the stimulatory effect of suboptimal insulin concentration on insulin receptor autophosphorylation. These results, together with our observations that the H_2_O_2 _scavenger (NAC) inhibited both insulin-stimulated H_2_O_2 _generation and insulin receptor autophosphorylation, suggest that the mitochondrial respiratory chain is involved in insulin-stimulated H_2_O_2 _production, thus playing an integral role in insulin receptor autophosphorylation in neurons.

Our prior studies provide evidence that a transient activation of succinate dehydrogenase (SDH) is a mode by which insulin increases the rate of mitochondrial H_2_O_2 _generation [20,21]. Earlier, it has been demonstrated that insulin exhibits an immediate stimulatory effect on oxidation of [2,3-^14^C]-succinate in mitochondrial Krebs cycle, which is almost maximal within 30 sec [38,39]. The results of the present study are consistent with these findings. Malonate, a competitive inhibitor of succinate dehydrogenase, inhibits both insulin-stimulated H_2_O_2 _production and the receptor phosphorylation in neurons, indicating a role of SDH in these processes. Although signaling pathways regulating insulin-stimulated SDH activation remain to be elucidated, these pathways seem to be distinct from those induced by autophosphorylated form of insulin receptor. The reason for it is that insulin-stimulated H_2_O_2 _burst enhances the autophosphorylation of the insulin receptor, since inhibitors of insulin-stimulated H_2_O_2 _generation abolish the receptor autophosphorylation.

A large body of evidence has accumulated that impairments in cerebral insulin receptor signaling may contribute to age-related cognitive decline and Alzheimer's disease [40-43]. In this context, our findings identify mitochondrial respiratory chain as a potential pharmacological target for the treatment of disorders associated with dysfunctional insulin receptor signal transduction in neurons.

## Conclusion

Results of the present study suggest that insulin-induced H_2_O_2 _is required for the enhancement of insulin receptor autophosphorylation in neurons. The mitochondrial respiratory chain is involved in insulin-stimulated H_2_O_2 _production, thus playing an integral role in insulin receptor autophosphorylation in neurons.

## Methods

### Materials

PhosphoDetect™ Insulin Receptor (pTyr1162/1163) ELISA kit and Insulin Receptor (β-Subunit) ELISA Kit were from Calbiochem. Dicholine salt of succinic acid was prepared by a reaction of succinic acid with choline base in the Russian Scientific Center on Drug Safety (Staraya Kupavna, Moscow region). Other materials were purchased from Sigma, ICN, Gibco, Biosource, Invitrogen, or Acros.

### Neuronal culture

Cerebellar granule neurons were prepared from 7- to 8-day-old Wistar rats as described [44,45]. Cerebellum was dissected and placed in ice-cold Ca^2+^/Mg^2+^-free Hanks' buffered salt solution (HBSS) without Phenol Red (Gibco). After mincing the tissue with fine scissors, the tissue was placed in Ca^2+^/Mg^2+^-free HBSS with Phenol Red and 0.1% trypsin for 15 min at 36°C. Trypsin was inactivated by washing with normal HBSS. Cells were dissociated by trituration and pelleted in HBSS. Then, the cells were resuspended in Neurobasal Medium (Gibco) supplemented with B-27 Supplement (Gibco), 20 mmol/L KCl, GlutaMax (Gibco) and penicillin/streptomycin and plated with density 5 × 10^6 ^cells/ml onto 35 mm × 10 mm sterile cell culture dishes which had been previously coated with poly-D-lysine. The cultures were maintained at 36°C in a humidified atmosphere of 5% CO_2 _and 95% air and fed with supplemented Neurobasal Medium. Cultures were treated on day 3 with 10 μmol/L cytosine arabinoside (Sigma) for 24 h to prevent glial proliferation. Neurons at 7 to 9 days were used for experiments.

### Measurement of hydrogen peroxide

H_2_O_2 _release from CGN cultures for 1 min was measured fluorimetrically employing the cell-impermeable Amplex Red dye (Invitrogen) in the presence of horseradish peroxidase (Sigma). With Amplex Red, it is possible to perform reliable measurements of H_2_O_2 _production by brain mitochondria under physiologically realistic conditions [46]. CGN cultures were pre-incubated for 30 min in Hepes-buffered salt solution (145 mmol/L NaCl, 5.6 mmol/L KCl, 1.8 mmol/L CaCl_2_, 1 mmol/L MgCl_2_, 20 mmol/L HEPES, and 5 mmol/L glucose) at pH 7.4 and then exposed to insulin (100 nM) or vehicle. H_2_O_2 _release from CGN cultures for 1 min was measured. Where indicated, NAC (5 mmol/L) was added 30 min before the insulin stimulation. Malonate (2 mmol/L) or FCCP (0.5 μmol/L) were added 5 min before the insulin stimulation. In these experiments, the incubation medium was supplemented with 2 μmol/L Amplex Red and 4 IU/ml horseradish peroxidase. Fluorescence was measured with an epifluorescent inverted microscope Axiovert 200 (Carl Zeiss, Germany) equipped with a 20× fluorite objective using excitation at 550 ± 10 nm and fluorescence detection at 610 ± 30 nm. All imaging data were collected and analyzing using the Metafluor 6.1 software (Universal Imaging Corp., USA). Standard curves obtained by adding known amounts of H_2_O_2 _to the assay medium were linear up to 1500 nmol/L. Fluorescence values were converted to H_2_O_2 _values using these standard curves. The calculated detection limit of the assay was 7 nmol/L. Data were normalized by cell density and expressed as nmol/L H_2_O_2_.

### Insulin receptor phosphorylation assay

Amounts of double phosphorylated β-subunit of insulin receptor (pYpY-IR) were measured by PhosphoDetect™ insulin receptor (pTyr1162/1163) ELISA kit (Calbiochem) suitable for studies with rat insulin receptor. CGN cultures were pre-incubated for 30 min in Hepes-buffered salt solution (145 mmol/L NaCl, 5.6 mmol/L KCl, 1.8 mmol/L CaCl_2_, 1 mmol/L MgCl_2_, 20 mmol/L HEPES, and 5 mmol/L glucose) at pH 7.4 and then exposed to insulin (100 nM) or vehicle for 20 min (the incubation time and the insulin concentration were determined from a time and dose response curves respectively; data not shown). Where indicated, NAC (5 mmol/L) was added 30 min before the insulin stimulation. Malonate (2 mmol/L) or FCCP (0.5 μmol/L) were added 5 min before the insulin stimulation. The experiment was terminated by removing the medium, washing with ice-cold PBS, and adding 120 μL per dish cell lysis buffer (Biosource) supplemented with 1 mmol/L PMSF, 50 mmol/L protease inhibitor set III (Sigma), and 2 mmol/L sodium ortovanadate as the inhibitor of tyrosine phosphatases at 4°C for 20 min. Lysates were centrifuged at 12,000 rpm at 4°C for 12 min. In each CGN lysate, pYpY-IR amounts were measured as described by the manufacturer's manual. Obtained values were normalized to total amounts of insulin receptor β-subunit (IR) measured by insulin receptor (β-subunit) ELISA kit (Calbiochem). The results are expressed as a percentage of the response produced to 100 nmol/L insulin.

### Statistics

Data were analyzed for statistical significance by one-way analysis of variance (ANOVA). Values are given as means ± SD. Differences were considered significant at P < 0.05.

## Abbreviations

CGN, cerebellar granule neurons; CS, dicholine salt of succinic acid; FCCP, carbonyl cyanide-4-(trifluoromethoxy)-phenylhydrazone; HBSS, Hanks' buffered salt solution; HEPES, 4-(2-hydroxyethyl)-1-piperazineethanesulfonic acid; NAC, N-acetylcysteine; PBS, phosphate-buffered saline; PMSF, phenylmethylsulfonyl fluoride; PTEN, phosphatase and tensin homolog; PTPs, protein tyrosine phosphatases; SD, standard deviation; SDH, succinate dehydrogenase.

## Authors' contributions

TPS carried out the *in vitro *studies with CGN cultures and data analysis. YES carried out the *in vitro *studies with CGN cultures and data analysis. NAP carried out the *in vitro *studies with CGN cultures and data analysis. VGP participated in the design of the *in vitro *studies with CGN cultures, critical intellectual discussion, and manuscript evaluation/critique. IAP conceived, designed and coordinated the study, and drafted the manuscript. All authors read and approved the final manuscript.
